# Maximal radial distance for predicting extraprostatic extension of prostate cancer: a histopathological-radiological study

**DOI:** 10.1186/s40644-026-01011-1

**Published:** 2026-02-21

**Authors:** Fabio Porões, Antoine Nobile, Lucien Widmer, Julian Vidal, Jana Di Vincenzo, Hugo Najberg, Frederic Fer, Audrey El Kaïm, Curzio Rüegg, Johannes M. Froehlich, Carolin Reischauer, Harriet C. Thoeny

**Affiliations:** 1 Department of Medical and Surgical Specialties, Faculty of Science and Medicine, University of Fribourg, Fribourg, Switzerland; 2 Department of Radiology, Cantonal Hospital of Fribourg, Chemin des Pensionnats 2-6, Case postale, Fribourg, CH-1708 Switzerland; 3 Institute of Pathology, Promed Laboratoire Médical SA, Fribourg, Switzerland; 4 Department of Neuroscience and Movement Science, Faculty of Science and Medicine, University of Fribourg, Fribourg, Switzerland; 5 Myodata Team, Institute of Myology, Paris, France; 6 Neuromuscular Investigation Centre, Neuromuscular Physiology and Evaluation Laboratory, Institute of Myology, Paris, France; 7 Department of Oncology, Microbiology, and Immunology, Faculty of Science and Medicine, University of Fribourg, Fribourg, Switzerland; 8 Department of Urology, Inselspital, University of Bern, Bern, Switzerland

**Keywords:** Magnetic Resonance Imaging, Prostatic neoplasms, Extraprostatic extension, Neoplasm staging, Histopathological correlation

## Abstract

**Background:**

There is a lack of a reliable parameter for accurate prediction of extraprostatic extension (EPE) of prostate cancer on MRI. We introduce a new parameter for predicting EPE on MRI: the maximal radial distance (maxRADD). It corresponds to the largest diameter of a prostate cancer focus (PCF) perpendicular to the contact with the prostate pseudocapsule. We compared accuracy and reliability of maxRADD with the previously proposed maximal capsular contact length (maxCCL).

**Materials and methods:**

In a single-centre study, we retrospectively included all consecutive patients undergoing prostate MRI between October 2018 and December 2020, followed by radical prostatectomy. One uropathologist and four radiologists determined maxRADD and maxCCL for each PCF. Accuracy in predicting EPE was assessed using the area under the curve (AUC), with optimal sensitivity/specificity cutoffs determined by the Youden J index using the histopathological findings as the gold standard. Correlations between histopathological and MRI values of maxRADD and maxCCL were analyzed using the Pearson correlation coefficient (r). Inter-/intra-reader agreements were evaluated using intraclass correlation coefficients (ICC).

**Results:**

A total of 79 men (mean age ± standard deviation, 65 ± 6 years) were evaluated. Twenty-six (32.9%) patients had prostate cancer with EPE. On histopathology, there was no significant difference in the accuracy of predicting EPE between maxRADD and maxCCL (AUC, 0.92 vs. 0.91, *p* = 0.28). The optimal cutoffs were ≥ 10.7 mm (sensitivity: 70%; specificity: 90%) for maxRADD and ≥ 12.8 mm (sensitivity: 90%; specificity: 70%) for maxCCL. Pearson correlation showed a strong correlation for maxRADD values determined on MRI with their histopathological counterparts (all *r* ≥ 0.7) for all readers. On MRI, inter-/intra-reader agreements were good for both parameters but significantly higher for maxRADD than for maxCCL (ICC, 0.87 vs. 0.80, *p* < 0.001 and 0.90 vs. 0.88, *p* < 0.001, respectively).

**Conclusion:**

MaxRADD permits assessing EPE with good accuracy and shows higher reliability compared with maxCCL. Reliable preoperative prediction of EPE improves treatment planning, potentially reducing positive surgical margins and improving outcomes for patients with prostate cancer.

## Background

Accurate local staging is crucial for the prognosis and treatment planning of prostate cancer [1–3]. Extraprostatic extension (EPE) carries a worse prognosis due to a greater risk of positive surgical margins, biochemical recurrence, metastatic disease, and lower cancer-specific survival after radical prostatectomy [4–6]. Preoperative assessment of EPE impacts the surgical strategy and decision of neurovascular bundle sparing. Neurovascular bundle sparing procedures can reduce the rates of erectile dysfunction and incontinence [7]. However, this less aggressive surgery may result in biochemical recurrence and treatment failure, due to the higher risk of positive surgical margins [8].

Magnetic Resonance Imaging (MRI) has been shown to be superior to nomograms such as Partain tables which have traditionally been used for prostate cancer staging [9]. However, accuracy of MRI for predicting EPE of prostate cancer is limited and there is significant inter-reader variability [10–12]. A recent meta-analysis reported that breach of the prostate pseudocapsule with direct tumor extension and maximal capsular contact length (maxCCL) were the most predictive features of EPE [13]. Although highly specific, direct tumor extension is rarely seen and has a low sensitivity (23.7%) [13]. The sensitivity of maxCCL is higher but requires measuring the curved contact line between the prostate cancer focus (PCF) and the prostate pseudocapsule, making the parameter prone to inaccuracies [14]. Therefore, new parameters are urgently needed.

In this study, we introduce a new morphometric parameter for predicting EPE on MRI that, to our knowledge, has not been proposed before: the maximal radial distance (maxRADD). It corresponds to the largest diameter of a PCF perpendicular to the contact with the prostate pseudocapsule. We assume that a rectilinear measure (straight line) such as maxRADD can be more reliably determined than a curved measure like maxCCL. Similar to maxCCL, we expect that there is a correlation between maxRADD and EPE. Thus, the purpose of this study is to compare accuracy and reliability of maxRADD and maxCCL for predicting EPE. In a final step, feasibility of an algorithm combining both parameters to improve accuracy is also evaluated.

## Materials and methods

### Patient population

**Fig. 1:**
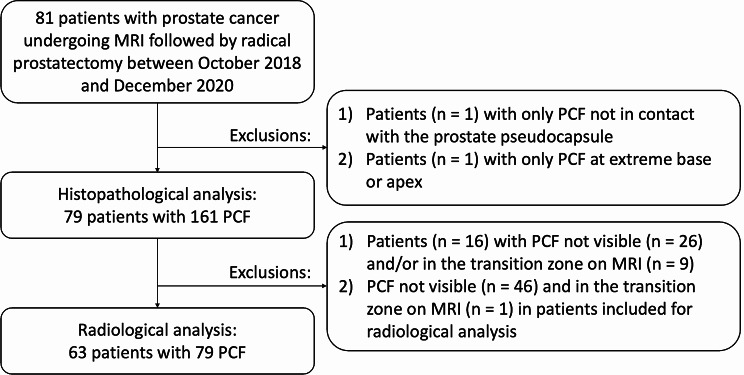
Flow diagram of the study population and selection of patients and prostate cancer foci (PCF) for histopathological and radiological analysis

This retrospective, single-centre study followed the Standards for Reporting Diagnostic Accuracy guidelines [15] and was approved by the Cantonal Ethical Committee of “Canton de Vaud” (BASEC no. 2020 − 01859) with a waiver for written informed consent. We included all consecutive patients who underwent prostate MRI at our institution between October 2018 and December 2020, followed by radical prostatectomy (Fig. 1). Exclusion criteria applying to the entire study population were prior prostate cancer treatment and MRI with insufficient image quality (PI-QUAL version 1 score < 4) [16]. For the histopathological analysis, PCF not in contact with the pseudocapsule, reaching the resection margin, or located at the extreme base or apex were excluded. For the radiological analysis only, PCF were further excluded if they were not visible on MRI or were located in the transition zone.

### Histopathological analysis

Radical prostatectomies were performed by five experienced surgeons with at least fifteen years of experience. Specimens were fixed with 4% buffered formaldehyde solution and entirely inked to allow assessment of surgical margins. The prostate was sectioned axially into 4 mm thick section slices in the plane perpendicular to the long axis of the prostate at midgland and sagittally at the base/apex. Due to the sagittal section plane, PCF at the extreme base/apex were excluded not allowing for measurement of maxCCL and maxRADD.

**Fig. 2:**
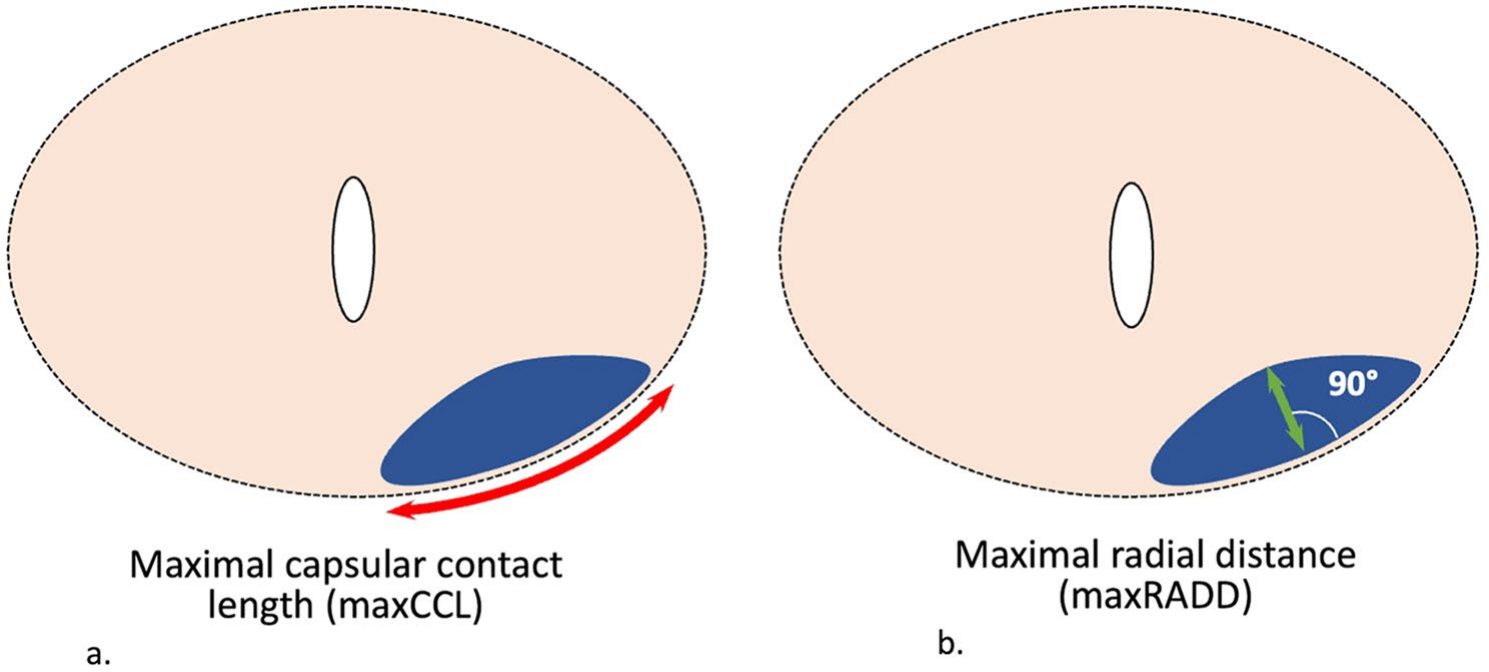
Measurement of (**a**) maximal capsular contact length (maxCCL) and (**b**) maximal radial distance (maxRADD)

**Fig. 3:**
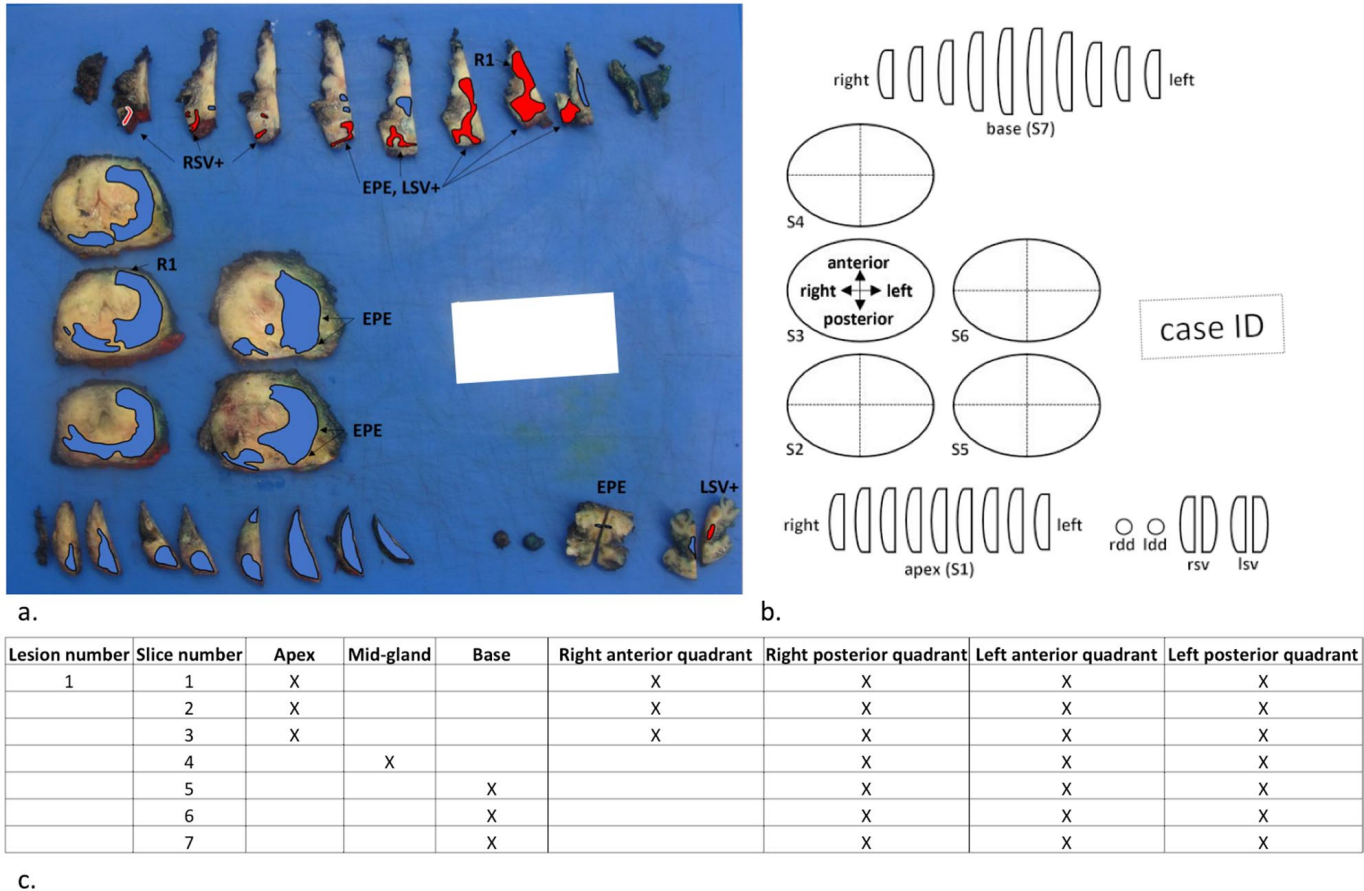
Prostate cancer focus (PCF) mapping: Macroscopic tumor cartography (**a**) and corresponding schematic representation (**b**) and diagram (**c**) showing a voluminous PCF with an ISUP (International Society of Urological Pathology) grade group of 2 and a Gleason score of 7 (3 + 4), extending from the base to apex and predominating on the left (blue) with extraprostatic extension (EPE), infiltration of the right seminal vesicle (RSV, red) and left seminal vesicle (LSV, red) and microscopic residual tumor residue (R1). rdd = right ductus deferens, ldd = left ductus deferens, S = slice

The histopathological analysis was performed by an uropathologist (A.N.) with 8 years of experience according to the International Society of Urological Pathology 2014 consensus statement [17]. He collected for each PCF: location, maxCCL, maxRADD, presence/absence of EPE, and length of EPE. Maximal radial distance was defined as the largest diameter of a PCF perpendicular to the contact with the pseudocapsule on axial slices (Fig. 2). The length of EPE was defined as the tumor extension perpendicular to the outer margin of the prostate [18]. PCF locations were mapped using a standardized template across three planes and reported on a diagram to allow histopathological-radiological correlation (Fig. 3). The histopathological findings served as the gold standard.

### MRI acquisition and image analysis

All patients underwent 3T MRI (Discovery MR750, GE Healthcare, Milwaukee, WI, USA) following Prostate Imaging Reporting and Data System version 2.1 [19–21]. Our prostate MRI protocol has been previously described [22].

All MRI examinations were initially interpreted by a senior radiologist with more than eighteen years of experience in prostate MRI (H.C.T). Finalised radiology reports were reviewed by four radiologists with 6 (Reader 1: J.V.), 3 (Reader 2: J.D.V.), 2 (Reader 3: F.P.) and 0 years (Reader 4: L.W.) of experience in prostate MRI who matched PCF initially reported with their histopathological counterparts using the PCF mapping diagram as reference. All four radiologists determined maxRADD and maxCCL only for PCF located in the peripheral zone on MRI. Prostate cancer foci in the transition zone were excluded as they were more difficult to correlate with confidence with histopathology and were less often in contact with the pseudocapsule [23].

Measurements were performed using Mint Lesion TM 3.0 software (Mint Medical GmbH, Heidelberg, Germany) enabling image cloning and independent evaluations. All MRI sequences were available to identify PCF, but maxRADD and maxCCL were determined on axial high b-value diffusion-weighted images (2000 s/mm^2^) and corresponding apparent diffusion coefficient maps as the dominant sequences for the detection of PCF in the peripheral zone according to Prostate Imaging Reporting and Data System version 2.1 [19–21]. Radiologists were aware of PCF location but blinded to EPE status. Prior to image assessment, all readers received a brief structured introduction to maxRADD, including five representative example cases to ensure a consistent measurement approach. No practice cases were performed. To assess intra-reader reliability, the analysis was repeated after three months in random order and blinded to the first reading.

### Statistical analysis

Analyses were performed by our statistical guarantors (H.N., F.F., A.E.K.) using R (R Foundation for Statistical Computing, Vienna, Austria) with a significance threshold of *p* < 0.05.

Pearson correlation coefficients assessed the association of MRI-derived maxRADD and maxCCL with their histopathological counterparts and with the length of the EPE [18].

Diagnostic performance was evaluated using sensitivity, specificity, receiver operating characteristic curve with area under the curve (AUC), and 95% confidence intervals (CIs) estimated via 10,000-iteration bootstrapping. Receiver operating characteristic curves were created with 100 thresholds ranging uniformly from the minimum to the maximum measured maxCCL and maxRADD. Non-inferiority of the AUC of maxRADD over maxCCL was tested using a t-test with an inferior bound of 10%. The Youden J index identified optimal sensitivity/specificity cutoffs using histopathological data [24]. Feasibility of an algorithm using both parameters was investigated to increase accuracy of predicting EPE.

Measurement reliability was assessed using intraclass correlation coefficients (ICC) [25]. Inter-reader agreement was evaluated using ICC (two-way random effects, single reader, absolute agreement) based on the first reading session. Intra-reader reliability was assessed using ICC (two-way mixed effects, single reader, absolute agreement) comparing the two reading sessions. Overall intra-reader reliability across all four readers was estimated with all evaluations pooled together. To compare ICCs, a bootstrap resampling procedure (1,000 iterations with replacement) was performed, and the distribution of ICC differences was analysed using a two-tailed t-test. According to Koo and Li, ICC values were classified as poor (< 0.5), moderate (0.5–0.75), good (0.75–0.9) or excellent (> 0.90) [26].

## Results

### Patient characteristics

A total of 81 consecutive patients underwent MRI of the prostate at our institution between October 2018 and December 2020 followed by radical prostatectomy. The process of patient and PCF inclusion is shown in Fig. 1. Our final study population for histopathological analysis consisted of 79 patients (mean age = 65.29 ± 6.8 years, range = 46–79 years) with 161 PCF. The baseline characteristics of our patients are provided in Table 1. Twenty-six (32.9%) patients had prostate cancer with EPE. Among the 161 PCF analysed, 27 (16.8%) showed EPE. After exclusion of PCFs not visible and/or in the transition zone on MRI, our population for radiological analysis consisted of 63 patients with 79 PCFs. The mean interval between MRI and surgery was 133.0 ± 85.6 days.

**Table 1:** Demographic and clinical characteristics of the study population (*n* = 79)

Variable	Value
Age (y)	65 (SD = 6, range = [46; 79])
Prostate-specific antigen (ng/mL)	10.4 (SD = 7.3, range = [3.7; 41.5])
Prostate volume at MRI (cm^3^)	43.9 (SD = 24.4, range = [14.1–152.0])
**ISUP grade group and Gleason score**:	
1, 3 + 3	3 (3.8)
2, 3 + 4	59 (74.7)
3, 4 + 3	7 (8.9)
4, 8	9 (11.4)
5, 9–10	1 (1.3)
**Histopathological EPE**:	
Present	26 (32.9)
Absent	53 (67.1)
**Histopathological SVI**:	
Present	11 (13.9)
Absent	68 (86.1)

Note.—Unless otherwise indicated, data are numbers of patients, and data in parentheses are percentages

EPE = extraprostatic extension, ISUP = International Society of Urological Pathology, SD = standard deviation, SVI = seminal vesicle invasion

### Histopathological-radiological correlation

Maximal radial distance and maxCCL measured on apparent diffusion coefficient maps demonstrated greater concordance with histopathological measurements compared to those obtained from diffusion-weighted images with a b-value of 2000 s/mm². The mean absolute difference between histopathological and radiological measurements was 1.29 ± 7.57 mm for apparent diffusion coefficient maps and 1.70 ± 7.38 mm for diffusion-weighted imaging. Consequently, apparent diffusion coefficient map-derived measurements were utilized for subsequent analyses.

Pearson correlation showed a strong correlation between both maxRADD and maxCCL determined on MRI with their histopathological counterparts (all *r* > 0.7), with the exception of maxCCL assessed by the reader without experience in prostate MRI (*r* = 0.54 for reading 1, 0.62 for reading 2) (Table 2). On histopathology, the mean value of maxRADD and maxCCL was 5.47 ± 3.99 mm and 10.48 ± 8.66 mm for PCF without EPE and 13.56 ± 4.73 mm and 37.20 ± 24.06 mm for PCF with EPE, respectively.

**Table 2:** Histopathological-radiological correlation of maximal capsular contact length (maxCCL) and maximal radial distance (maxRADD) Values

Reader	maxCCL		maxRADD	
	Reading 1	Reading 2	Reading 1	Reading 2
Reader 1	0.83	0.82	0.75	0.72
Reader 2	0.80	0.74	0.77	0.75
Reader 3	0.81	0.81	0.79	0.76
Reader 4	0.54	0.62	0.76	0.76

Note.—Data are r coefficients assessed by using Pearson correlation. maxCCL = maximal capsular contact length, maxRADD = maximal radial distance

### Diagnostic performance

**Fig. 4:**
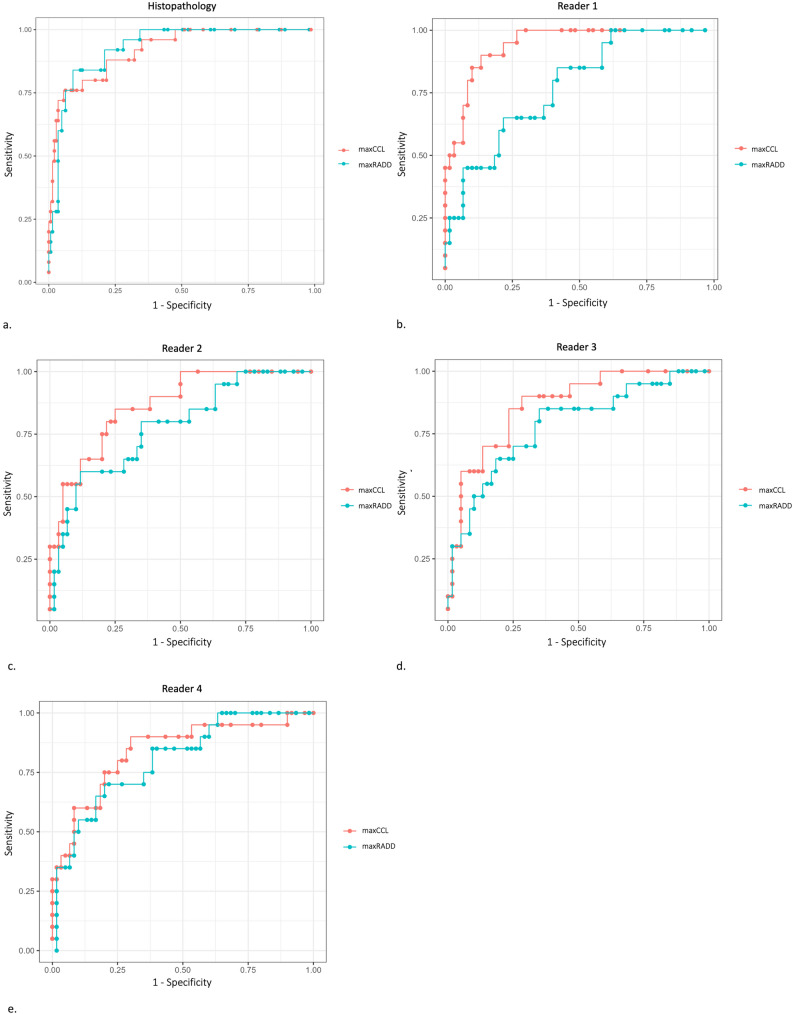
Receiver operating characteristic curves show performance of maximal capsular contact length (maxCCL) and maximal radial distance (maxRADD) when determined on (**a**) histopathology and on MRI by readers with different experience in prostate MRI: (**b**) reader 1 with 6 years of experience, (**c**) reader 2 with 3 years of experience, (**d**) reader 3 with 2 years of experience, and (**e**) reader 4 without experience

**Fig. 5:**
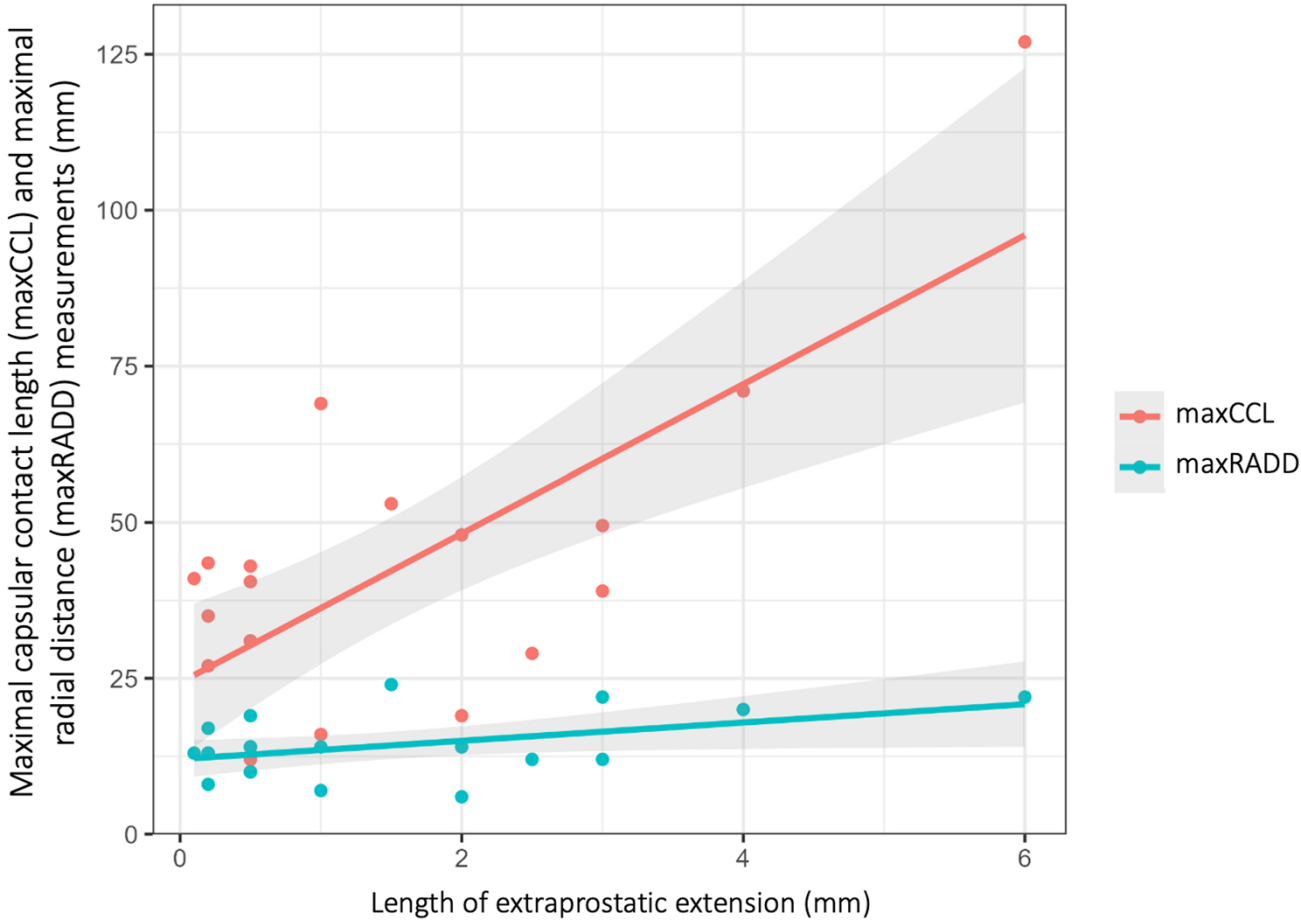
Correlation curves show significant linear correlations of maximal capsular contact length (maxCCL) and maximal radial distance (maxRADD) with length of extraprostatic extension. Shaded areas represent the 95% confidence intervals

Table 3 and Fig. 4 summarize the diagnostic performance of maxRADD and maxCCL determined on histopathology and on MRI by the four readers. On histopathology, there was no significant difference in the accuracy for predicting EPE between maxRADD and maxCCL (AUC_maxRADD_ = 0.92, 95% CI = [0.92, 0.92]; AUC_maxCCL_ = 0.91, 95% CI= [0.91, 1]; difference = 0.11, *p* = 0.28). On MRI, the AUC of maxCCL for prediction of histopathological EPE was significantly higher compared with the AUC of maxRADD, for reader 1 (AUC_maxRADD_ = 0.77, 95% CI = [0.76; 0.79]; AUC_maxCCL_ = 0.94, 95% CI = [0.93; 0.95], difference = 0.17, *p* < 0.001). The AUCs of the other readers were also higher for maxCCL, but did not reach significance. On histopathology, there was no significant difference in the correlation of both parameters with the length of histopathological EPE (Fig. 5).

**Table 3:** Diagnostic performance for histopathological extraprostatic extension

Reader	maxCCL AUC*	maxRADD AUC*	Difference	*p*-value
Histopathology	0.91 (0.91 ; 1)	0.92 (0.92 ; 0.92)	0.01	0.28
Reader 1	0.94 (0.93 ; 0.95)	0.77 (0.76 ; 0.79)	0.17	< 0.001
Reader 2	0.88 (0.75 ; 1)	0.77 (0.77 ; 0.79)	0.11	0.44
Reader 3	0.85 (0.84 ; 1)	0.78 (0.75 ; 0.78)	-0.08	0.07
Reader 4	0.83 (0.82 ; 1)	0.80 (0.78 ; 0.81)	-0.03	0.46

Note.—Data in parentheses are 95% confidence intervals

AUC = area under the receiver operating characteristic curve, maxCCL = maximal capsular contact length, maxRADD = maximal radial distance

* Receiver operating characteristic curves were created with 100 thresholds ranging uniformly from the minimum to the maximum measured maxCCL and maxRADD

For maxRADD, the optimal cutoff for the assessment of EPE was 10.7 mm or greater showing high specificity (122 of 134 [90%]) and moderate sensitivity (20 of 27 [70%]). For maxCCL, the optimal cutoff for the assessment of histopathological EPE was 12.8 mm or greater showing high sensitivity (25 of 27 [90%]) and moderate specificity (90 of 134 [70%]). Table 4 compares the diagnostic performance of these cutoff values with the two cutoff values established in the literature for maxCCL of 10 mm and 15 mm [13].

**Table 4:** Diagnostic performance for histopathological extraprostatic extension at defined cutoff values

Variable	Cutoff	AUC	Sensitivity (%)	Specificity (%)
**maxCCL**	≥10.0 mm*	0.86 (0.76 ; 0.89)	100 (78 ; 100) [26/27]	50 (45 ; 62) [72/134]
	≥12.8 mm†	0.88 (0.8 ; 0.92)	90 (74 ; 100) [25/27]	70 (59 ; 75) [90/134]
	≥15.0 mm*	0.88 (0.79 ; 0.92)	90 (71 ; 97) [24/27]	70 (64 ; 79) [96/134]
**maxRADD**	≥10.7 mm†	0.90 (0.83 ; 0.95)	70 (54 ; 88) [20/27]	90 (85 ; 95) [122/134]

Note.—Data in parentheses are 95% confidence intervals. Data in brackets are numerators and denominators

AUC = area under the receiver operating characteristic curve, maxCCL = maximal capsular contact length, maxRADD = maximal radial distance

* Established cutoffs in the literature [13]

† Optimal cutoffs selected with the Youden J index

**Fig. 6:**
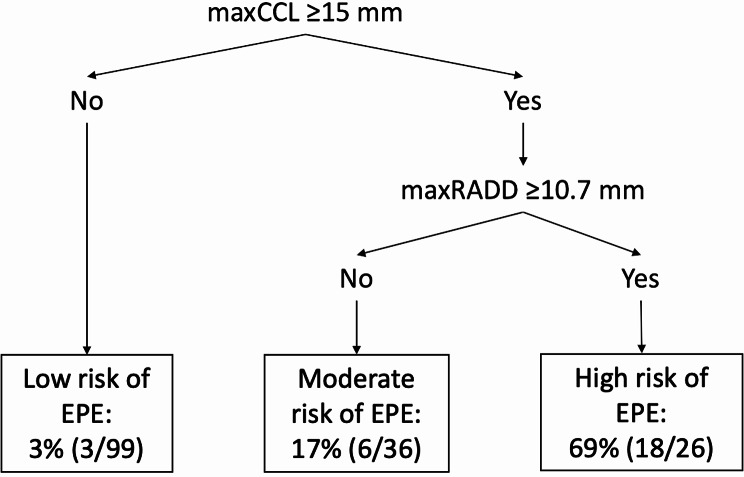
Algorithm for classifying the risk of histopathological extraprostatic extension (EPE) using the established cutoff of 15 mm for maximal capsular contact length (maxCCL) and the optimal cutoff of 10.7 mm for maximal radial distance (maxRADD). The percentages correspond to the proportion of PCF with an EPE on histopathology, with numerator and denominator in brackets

Using the established cutoff of 15 mm [13] for maxCCL also used in our clinical practice and the optimal cutoff of 10.7 mm for maxRADD, we developed a two-step algorithm to classify the risk of EPE into three categories as shown in Fig. 6. Due to the high sensitivity of maxCCL, it was used in the first step of the algorithm to detect PCF with a possible EPE. In the second step, the lack of specificity of maxCCL could then be compensated by the high specificity of maxRADD. Prostate cancer foci with a maxCCL of less than 15 mm had a low risk of EPE (3%). Prostate cancer foci with a moderate risk of EPE (17%) were defined as maxCCL of 15 mm or greater but maxRADD less than 10.7 mm. When PCF had both parameters at or above their respective cutoff values, they were at high risk of EPE (69%).

### Inter- and intra-reader agreement

**Fig. 7:**
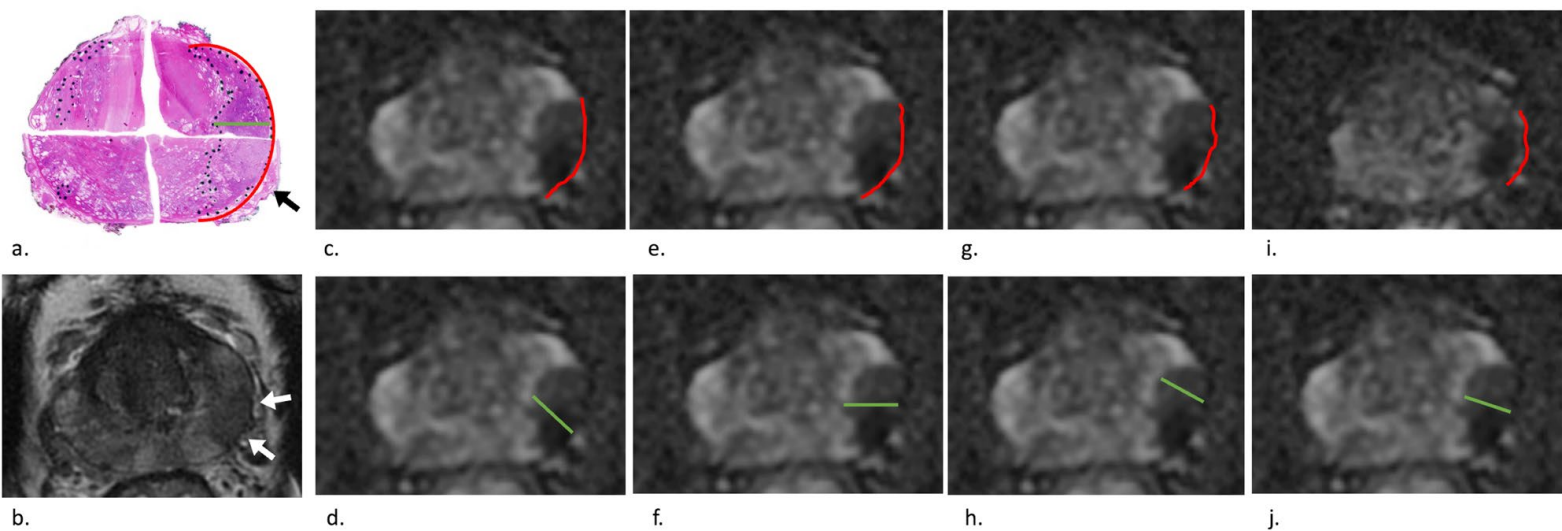
Images in a 68-year-old man who underwent radical prostatectomy for prostate cancer. (**a**) Step-section histopathological specimens show a prostate cancer focus (PCF) with an ISUP (International Society of Urological Pathology) grade group of 2 and a Gleason score of 7 (3 + 4), in the left peripheral zone (black dashed outline), with focal extraprostatic extension (EPE, black arrow), a maximal capsular contact length (maxCCL, red line) of 35 mm, and a maximal radial distance (maxRADD, green line) of 13 mm. (**b**) T2-weighted axial MRI image show the corresponding T2 hypointense tumor (white arrows) in the left peripheral zone and (**c-j**) apparent diffusion coefficient maps show measurements of maxCCL (red line) and maxRADD (green line) by the 4 readers: (**c-d**) reader 1 (maxCCL = 24 mm, maxRADD = 11 mm), (**e-f**) reader 2 (maxCCL = 25 mm, maxRADD = 12 mm), (**g-h**) reader 3 (maxCCL = 24 mm, maxRADD = 11 mm), and (**i-j**) reader 4 (maxCCL = 20 mm, maxRADD = 12 mm)

Inter-reader agreement was good for both parameters but significantly higher for maxRADD than for maxCCL (ICC_maxRADD_ = 0.87, 95% CI = [0.84; 0.89]; ICC_maxCCL_ = 0.80, 95% CI = [0.75; 0.84], difference = 0.07, *p* < 0.001, representative example in Fig. 7). Intra-reader agreement was good for both parameters but significantly higher for maxRADD (pooled ICC_maxRADD_ = 0.90, 95% CI = [0.88; 0.91], pooled ICC_maxCCL_ = 0.88, 95% CI = [0.86, 0.89], difference = 0.02, *p* < 0.001). The ICC values representing inter- and intra-reader agreements are summarized in Table 5.

**Table 5:** Intra- and interreader agreement for radiological assessment of maximal capsular contact length (maxCCL) and maximal radial distance (maxRADD)

Variable	maxCCL	maxRADD	Difference	*p*-value
Interreader Agreement	0.80 (0.75 ; 0.84)	0.87 (0.84 ; 0.89)	0.07	< 0.001
Intrareader Agreement	0.88 (0.86 ; 0.89)	0.90 (0.88 ; 0.91)	0.02	< 0.001
Reader 1	0.92 (0.89 ; 0.94)	0.91 (0.89 ; 0.93)	0.01	0.222
Reader 2	0.87 (0.83 ; 0.90)	0.91 (0.89 ; 0.93)	0.04	< 0.001
Reader 3	0.97 (0.95 ; 0.97)	0.92 (0.90 ; 0.94)	0.05	< 0.001
Reader 4	0.70 (0.61 ; 0.76)	0.86 (0.81 ; 0.89)	0.16	< 0.001

Note.— Data are intraclass correlation coefficients (ICC), and data in parentheses are 95% confidence intervals

maxCCL = maximal capsular contact length, maxRADD = maximal radial distance

## Discussion

Predicting EPE on MRI is a critical step in clinical management of prostate cancer and may result in better oncologic and functional outcomes after surgery. In this study, we introduce maxRADD, the largest diameter of a PCF perpendicular to the contact with the prostate pseudocapsule, as a new parameter for predicting EPE on MRI. Based on histopathological and radiological measurements, we compared accuracy and reliability of maxRADD with the previously proposed maxCCL for predicting EPE. On histopathology, accuracy of maxRADD and maxCCL in predicting EPE was equivalent (area under the curve [AUC], 0.92 vs. 0.91, *p* = 0.28). On MRI, performance tended to be higher with maxCCL and increased with increasing reader experience. However, performance was only significantly higher for the reader with the most experience. This observation may reflect a combination of factors, including greater reader familiarity with maxCCL, differences in the analysed PCF between histopathological and radiological assessments, as well as intrinsic differences in how these parameters translate from histopathological specimens to MRI images. While all PCF were included in the histopathological analysis, the radiological analysis was restricted to MRI-visible peripheral zone PCF, which may partly explain discrepancies in diagnostic performance. In addition, the lower spatial resolution of MRI compared with histopathology may differentially affect these measurements, with maxRADD potentially being more sensitive to small inaccuracies in PCF boundary delineation. Reliability was significantly higher for maxRADD as shown by inter- and intra-reader agreements (intraclass correlation coefficients [ICC], 0.87 vs. 0.80, *p* < 0.001 and 0.90 vs. 0.88, *p* < 0.001, respectively).

To the best of our knowledge, the use of maxRADD has not previously been established in the literature. Conversely, the use of maxCCL to predict EPE is well established and our results are in line with the literature. A recent meta-analysis [13] estimated sensitivity at 86.3% and specificity at 62.5% for maxCCL at a cutoff of 10 mm, rather close to our results (sensitivity = 100% and specificity = 50%). With a cutoff value set at 15 mm for maxCCL, Mehralivand et al. [6] found a sensitivity at 70% and specificity at 72% also consistent with our results (sensitivity = 90% and specificity = 70%). The slight difference in results can be explained by the fact that we used maxCCL measurements performed in histopathology as our gold standard. Although parameters had similar accuracy, they were opposite in terms of sensitivity and specificity. Using the high sensitivity of the established cutoff of 15 mm for maxCCL also used in the “EPE grade” developed by Mehralivand et al. [6], as well as the high specificity of the optimal cutoff of 10.7 mm in our study, we proposed an algorithm to classify PCF according to their risk of EPE and improve overall accuracy. Our moderate risk for EPE defined as maxCCL greater than 15 mm and maxRADD less than 10.7 mm corresponds to an “EPE grade” 1 with a similar probability of histopathological EPE (24% vs. 17%). Prostate cancer foci exceeding both thresholds are considered high risk, closely matching the “EPE grade” 3 group (66% vs. 69%). Maximal radial distance reflects the growth of a PCF in an orientation perpendicular to the prostate pseudocapsule and can be seen as a quantitative representation of qualitative parameters such as the bulging/irregularity and breach of the prostate pseudocapsule assessed in the “EPE grade” [6].

Park et al. [18] reported substantial inter-reader agreement with a Cohen κ of 0.74 and intrareader agreement with a weighted κ of 0.74 for maxCCL ≥10 mm, superior to subjective assessment and semi-quantitative scores (interreader agreement: 0.63–0.71 and intrareader agreement 0.61–0.69). The lower reliability for maxCCL reported by Park et al. compared to the current study can be explained by the fact that we did not set a threshold for maxCCL for this analysis. In accordance with other publications, our results suggest an improved reliability with quantitative parameters compared to qualitative/subjective assessment [18, 27]. The distinction between irregularity, bulge and breach of the prostate pseudocapsule is not always easy, especially for inexperienced readers and can lead to a decrease in reliability [18]. In the current study, maxRADD showed good reliability and may be an alternative for assessing these qualitative parameters. In addition, we can expect better reliability of our algorithm combining the two parameters for the assessment of EPE, including only quantitative parameters compared to a semi-quantitative score such as the “EPE grade”. This algorithm is of real clinical interest, as it is easy to use and teach, and could provide a reliable and consistent analysis of EPE on MRI. It could also offer significant improvements over the two parameters used on their own, and potentially replace the “EPE grade”. Nevertheless, the proposed two-step algorithm is exploratory and derived from a single dataset. Independent validation is required before any clinical implementation. An internal validation study is underway to evaluate the performance and reliability of this algorithm and compare it to the “EPE grade”.

This study has several limitations. First, PCF on sagittal slices at the base/apex were excluded not allowing for measurement on histopathology. In addition, the use of whole-mount histopathological sections could further improve spatial correspondence between histopathology and MRI, potentially strengthening histopathological-radiological correlations in future studies [28]. The axis of the radical prostatectomy sections is not strictly equivalent to the strict axial slices of the MRI, which may have had an influence on the values of the two parameters assessed. These shortcomings must have had a limited effect on our results, given the good correlation between both parameters determined on MRI with their histopathological counterparts (all *r* ≥ 0.7), when assessed by readers with experience in prostate MRI. Our cutoff value for maxRADD was established using histopathology values, although it is known that tissue shrinks after formalin fixation. Considering that our optimal cutoff value for maxCCL was 12.8 mm, exactly between the two cutoff values established in the literature of 10 mm and 15 mm, and that linear shrinkage is estimated at only 4.5% [29], we did not correct our cutoff value for maxRADD. Finally, 82 PCF were excluded from the radiological analysis either because they were not visible on MRI or because they were located in the transition zone. Prostate cancer foci in the transition zone were excluded because they are more difficult to identify and less often in contact with the prostate pseudocapsule [23]. Focusing on peripheral zone PCF allowed consistent measurements on diffusion-weighted images and corresponding apparent diffusion coefficient maps, avoiding sequence-related bias that would arise from T2-based measurements in the transition zone, and targeted the clinically most relevant subgroup for MRI-based EPE prediction, as these PCF are more prevalent and more frequently in contact with the pseudocapsule [23]. Nevertheless, this selection could have led to an overestimation of radiological performance and reliability. To address this limitation, an internal validation study is currently ongoing in which transition zone PCF will also be included in the radiological analysis.

Beyond these methodological considerations, the generalizability of our results should be interpreted with caution. This was a single-centre, retrospective study with a relatively limited number of PCF with positive EPE (27/161, 16.8%), which may limit external validity. In addition, institutional MRI acquisition practices may influence external validity. Focused prostate MRI sequences can be acquired either in a strict axial plane or aligned with the prostate axis. In our institution, strict axial acquisitions are routinely used, as they provide, in our experience, better measurement reproducibility. However, variations in acquisition planes across centres could lead to slightly different measurements and results. Measurements were performed on diffusion-weighted images and corresponding apparent diffusion coefficient maps as the dominant sequences for the detection of PCF in the peripheral zone according to Prostate Imaging Reporting and Data System version 2.1 [19–21] and because our institution benefits from very high-quality diffusion imaging (3T acquisition, use of multiple b-values, optimized patient preparation) [22, 30]. Diffusion image quality may vary across centres, potentially limiting measurement performance and reproducibility in settings with lower-quality diffusion-weighted images. As part of our ongoing internal validation, we plan to assess maxRADD and maxCCL measurements across all MRI sequences, including T2-weighted images, diffusion-weighted images and corresponding apparent diffusion coefficient maps and dynamic contrast-enhanced images. Finally, reader training for maxRADD was limited to the review of a small number of example cases without hands-on practice. More extensive preparation, including practice cases, could further improve both diagnostic performance and measurement reliability of maxRADD across readers.

## Conclusion

We propose a new quantitative parameter for predicting extraprostatic extension (EPE) on MRI: the maximal radial distance (maxRADD). Compared with the previously proposed maximal capsular contact length (maxCCL) it has an equivalent accuracy for predicting EPE but higher reliability. Combining the two parameters, we propose a two-step classification algorithm for the risk of EPE into low, moderate and high risk. This algorithm should be relatively simple to implement as it is based solely on two quantitative parameters, which makes it easy to teach and use, and as it provides a quantifiable and graded assessment of the risk of histopathological EPE. We are currently evaluating its performance in a separate internal study. External validation studies are needed to assess the generalisability of our promising results.

## Data Availability

The datasets analyzed for the current study are not publicly available due to patient privacy. The data will be shared upon reasonable request by the corresponding author.

## Abbreviations

AUC: Area under the curve
CI: Confidence interval
EPE: Extraprostatic extension
ICC: Intraclass correlation coefficients
maxCCL: Maximal capsular contact length
maxRADD: Maximal radial distance
MRI: Magnetic Resonance Imaging
PCF: Prostate cancer focus
